# Investigation of an Outbreak of Diphtheria in Borborooah Block of Dibrugarh District, Assam

**DOI:** 10.4103/0970-0218.69282

**Published:** 2010-07

**Authors:** Benu Nath, Tulika Goswami Mahanta

**Affiliations:** Department of Community Medicine, Assam Medical College, Dibrugarh, Pin - 786002, India

## Introduction

Diphtheria is a highly contagious and potentially life threatening bacterial disease caused by corynebacterium diphtheria.(1) The EPI of WHO recommends three doses of DPT vaccine starting at six weeks of age with additional doses of diphtheria vaccine in countries where resources permit. Many national immunization programs, including the UIP in India offer two booster doses at 18 months and between 54 to 72 months of age; after three doses of primary vaccines, protective levels of antitoxin develop in 94–100% of the children. However without booster doses, over time toxoid induced antibody drops below protective level.(2)

In 2008, India contributed 6081(86.66%) of the 7017 diphtheria cases reported globally.(3) There were no reports of outbreaks of diphtheria in Assam since last few years, though sporadic cases were reported in UIP monthly report, which were never investigated and documented. The number of cases coming to Assam Medical College was very few and the immunization coverage in Assam was 19.30% in 2006(RHS), which has improved to 67.60% in 2006–2007. In Dibrugarh District of Assam in 2008–2009, administrative data shows coverage of 90%, while the evaluated data from Regional Resource Centre shows coverage of 78%. As outbreak of diphtheria reflects the impact of immunization outbreak investigation was carried out to assess the diphtheria outbreak pattern and case fatality rate in Borborooah block of Dibrugarh district of Assam.

## Materials and Methods

Outbreak investigation was done with the use of operational definition.(4) All epidemiological data were collected. Active search of cases was done by doing house-to-house survey in all the villages by ANM and ASHAs, after imparting training on collection of demographic information, line listing of cases. Treatment protocol was implemented from the second week with erythromycin tab through ANMs, for all the symptomatic cases. Community-wise awareness programs were carried out regarding respiratory etiquette, use of homemade musk, hygiene and cleaning of households. Health workers were also trained to protect themselves by use of musk, hand hygiene. Early diagnosis and prompt treatment protocol was implemented. Laboratory investigation was done in 44 symptomatic cases, where the C. diphtheria shows sensitivity to erythromycin.

## Results

A total of 60 cases of diphtheria were reported. Majority of cases(40%) belonged to 20–44 years of age group, while 6.66% belonged to 0–4 years of age. Males are affected more than females which are 53.33% and 46.67% respectively [Table 1]. Out of these 60 cases which were epidemiologically linked, laboratory investigation was done in 44 cases and lab confirmed cases were eight in numbers(18.18%). Of the eight confirmed cases, 62.5% were ≥ 20 years of age group, 37.5% were 10–19 years of age group. In addition, five cases were males(62.5%) and three cases were females(37.5%).

**Table 1:** Distribution of diphtheria cases by age and sex

Age in years	Sex (lab confirmed)		Total	Percentage
	Male	Female		
0–4	4 (0)	0 (0)	4	6.66
5–9	6 (0)	1 (0)	7	11.67
10–14	5 (2)	7 (1)	12	20
15–19	3 (0)	5 (0)	8	13.33
20–44	11 (3)	13 (2)	24	40
≥45	3 (0)	2 (0)	5	8.33

Total	32 (5)	28 (3)	60	100

**Figure 1:**
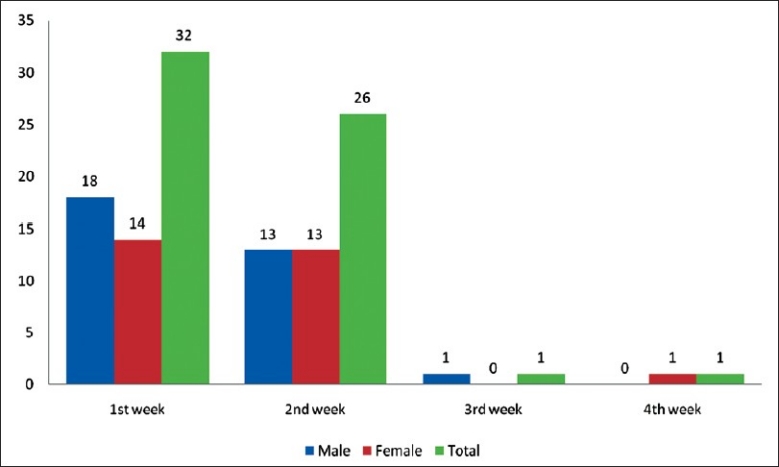
Weekly sex-wise distribution of diphtheria cases

Figure 1 shows that maximum(53.33%) numbers of cases were found during the first week of outbreak of which 30% were males and 23.33% were females. 43.34% of cases were found in second week with equal male to female ratio, thereafter cases started declining reaching zero level at the end of fourth week. Out of these 60 cases of diphtheria, two cases died(case fatality rate -3.33%). The first and second case of this outbreak were the only fatal cases, where there was delay in getting treatment and referral. Immunization coverage of the Borborooah block of Dibrugarh district of Assam during the year 2008–2009 was as follows: BCG-82%, DPT1-82%, DPT3-77%, Measles-71%. Fully immunized 69% with a dropout rate of DPT1 and DPT3 is 6%.

## Discussion

In the past, diphtheria was considered as one of the most serious childhood diseases because it took a heavy toll on health and life of preschool aged children. Prior to the widespread availability of diphtheria toxoid, nearly 70% of cases were children younger than 15 years of age. With the advent of EPI in 1978 and UIP in 1985, most of the VPDs have shown a decline but diphtheria is still endemic in our country.(5)

Although diphtheria is a childhood VPDs, in this study it has been found that maximum cases are of 20–44 years age group. Sailaja Bitragunta *et al* (in a Hyderabad-based study) also obtained almost similar result,(6) which may indicate low immunization coverage in last few decades before the launch of NRHM. Such type of natural distribution of disease may also suggest the importance of adolescent immunization with diphtheria vaccine. Similar to other studies, this study also shows more number of male cases,(7) but in a study carried out in a rural medical college near Kolkata, they found no sex differences in diphtheria cases.(8) Khan *et al* in their study on “resurgence of diphtheria in vaccination era” observed that females were mostly affected than males.(5)

In the present study, diphtheria was diagnosed mainly on clinical findings and confirmed by epidemiological linkage with lab confirmed cases and microbiological confirmation was available in 18.18% of cases. Ray *et al*.(9) (in their study conducted in rural medical college hospital near Kolkata) also observed the low microbiological confirmation rate and suggested that that clinical diagnosis of diphtheria should be given due consideration.(9)

Diphtheria outbreak occurs in the month of July to August in our study. Several studies(7–9) carried out over the last 30 years at different places in this country also reported that diphtheria occurs more frequently during the month of August to November, which may be because of early monsoon in North eastern region. In this study, it was evidenced that early diagnosis and prompt treatment can reduce fatality. The case fatality rate was found to be almost similar, consistent with the findings of Kadirova *et al*.(10) while, diphtheria outbreak in Cali, Colombia, August-October 2000, reported case fatality rate as 12.5%.(11)

In Assam, the percentage of children of 12-23 months who have received all the vaccines as found in NFHS III was 31.7 in rural and 29.3 in urban areas. Although immunization drive conducted by NRHM has improved the administrative data, and was also reflected by coverage evaluation done by independent agency; strengthening routine immunization is a long-term proposition and we should demand steady progress but not expect giant leaps.(12,13)

## Conclusion

More morbidity and mortality among older age group may reflect poor immunization coverage in last few decades against diphtheria and also waning immunity. This is a matter of concern for public health. It was evidenced from this study that early diagnosis and active treatment of cases have the potential to reduce the CFR. A good surveillance system is essential to detect the possible outbreak of diphtheria as early as possible.
